# Primary Healthcare Center’s Healthcare Providers’ Perception and Practice Toward Pediatric Cow's Milk Allergy in Qassim Region, Saudi Arabia

**DOI:** 10.7759/cureus.41719

**Published:** 2023-07-11

**Authors:** Yazeed A Alghasham, Aeshah M Alharbi, Kadi A Alhumaidi, Yasir S Alkhalifah

**Affiliations:** 1 Department of Pediatrics, Unaizah College of Medicine and Medical Sciences, Qassim University, Unaizah, SAU; 2 College of Medicine, Unaizah College of Medicine and Medical Sciences, Qassim University, Unaizah, SAU; 3 Department of Pediatrics, Majmaah University, Riyadh, SAU

**Keywords:** cow’s milk allergy, primary healthcare center, practice, knowledge, healthcare provider

## Abstract

Background: Cow's milk allergy (CMA) typically first manifests in the first year of life, and it is the most challenging food allergy to detect since the clinical symptoms can vary significantly in both types and severity. This study is carried out to evaluate the level of knowledge and practice of healthcare providers (HCPs) in the Qassim Region regarding CMA.

Research methodology: This is a cross-sectional study conducted among HCPs in the Qassim Region, Saudi Arabia. A self-administered questionnaire was distributed among HCPs using face-to-face interviews compromising four governorates of Qassim such as Buraydah, Unaizah, AlRass, and Albukairyah. The questionnaire includes socio-demographic characteristics and questions to assess the knowledge and practice of HCPs regarding CMA.

Results: Among 124 HCPs, 29% were aged between 25 and 30 years, 50.8% were males and 49.2% were females. Over three-quarters (77.4%) were categorized as having poor knowledge levels, while poor practice was also prevalent (83.1%). Factors associated with increased knowledge and practice were being non-Saudi, being a consultant/specialist, and having more than 10 years of experience. There was a significant positive correlation between knowledge and practice scores (p<0.001).

Conclusion: The knowledge and practice of HCPs regarding CMA were insufficient. Non-Saudi consultants/specialists with more years of experience tend to be more knowledgeable and have better skills in managing the disease. Further longitudinal studies are required to establish the level of knowledge and practices toward CMA.

## Introduction

Cow's milk allergy (CMA) typically first manifests in the first year of life, affecting approximately 2% to 3% of children under four years of age, and it is the most challenging food allergy to detect since the clinical symptoms can vary greatly in both types and severity [1]. Therefore, it is one of the most prevalent food allergies in children, along with egg and peanut allergies, and like other food allergies, its incidence is expected to increase [2,3]. The primary symptoms are cutaneous manifestations, followed by gastrointestinal and respiratory manifestations in the majority of patients. A life-threatening anaphylactic reaction may also occur in 12% of cases [4].

Different immunological mechanisms can cause CMA; the IgE-mediated pathway, which is activated by IgE immunoglobulins, accounts for roughly 60% of cases. Symptoms of this form of allergy start to manifest within two hours. The non-IgE-mediated reaction form of CMA, which manifests between three hours and one week after ingesting the allergenic proteins, is characterized mostly by gastrointestinal symptoms [4]. The absolute elimination of cow's milk from the child's and mother's diet is recommended as part of a restriction diet, along with the use of suitable cow's milk substitute formulas such as extensive hydrolyzed formula and amino acid formula [5,6]. Moreover, there are recommendations to breastfeed an infant solely for four to six months and to delay the introduction of exogenous proteins and solid foods [1,7,8]. It's also important to distinguish between CMA and lactose intolerance (LI) since they have the same symptoms but with different mechanisms [1,7]. While rapid IgE-mediated reactions, in general, are immediately recognized, the diagnosis of non-IgE-mediated CMA may be difficult due to the non-specific character of gastrointestinal symptoms that overlap with typical non-allergic juvenile diseases [9]. Healthcare providers' (HCPs) limited awareness of non-IgE-mediated CMA may result in a delayed or inaccurate diagnosis as well as inadequate nutritional therapies that may worsen nutritional outcomes [10,11].

According to a cross-sectional study that was conducted to assess the knowledge and practice of healthcare professionals regarding the management of CMA, it is found that there are misconceptions and knowledge gaps about the diagnosis and therapeutic management of CMA. However, the symptoms and signs of IgE-mediated CMA were well-known to healthcare practitioners [12]. Moreover, another long-term study that was carried out in Turkey among primary-care physicians shows that knowledge of food allergy was unsatisfactory and revision or periodic educational programs should be aimed at improving the standard of practice as acknowledged by the participants [13].

While we were searching about this topic, we noticed insufficient studies assessing HCPs' knowledge and their practice toward CMA in Saudi Arabia. In our study, we aim to assess it by developing a survey that targets HCPs. According to our knowledge, there is no previous study to assess this matter in the Kingdom of Saudi Arabia.

## Materials and methods

This cross-sectional study was conducted in the Qassim Region of Saudi Arabia from 8 December 2022 to 15 March 2023 in which primary HCPs participated. According to the Saudi statistical yearbook 2017, the Qassim Region contains 181 primary healthcare centers [14]. However, the study was conducted in the four governments of the Qassim Region out of 14 (Buraydah, Unaizah, AlRass, and Albukairyah). The selection of governments was according to the population and the number of primary healthcare centers. In the four chosen governments, the total number of primary healthcare centers was 86. On average, each center usually has two general physicians. Therefore, the total number of physicians became 172 which served as a population for sample size calculation. To calculate the sample size for the study, OpenEPi was used. The total population size was 172, the chances of inclusion of any physician were 50%, and the confidence interval was set as 95%. Hence, by using the simple random sampling method, the calculated sample size for the study was 119.

To collect the required sample size, a convenience sampling method was used. From each of the selected governments, 50% of the primary healthcare centers were selected, and physicians working in those centers were invited to participate in the study. Physicians of both genders could participate in the study. Any health physician working outside the selected government was excluded from the study.

A self-administered questionnaire was devised for the data collection by the study authors. The native language of the study authors was Arabic; therefore, the questionnaire was first prepared in Arabic and then translated into English. An English language professional was requested for the translation. The English version was translated back into Arabic to verify that the English version contains the actual meaning. After the translation, a pilot study was conducted to validate the questionnaire.

The knowledge of the HCPs regarding CMA was assessed using a four-item questionnaire, where the correct answers are identified and coded with 1 while the incorrect answer is coded with 0. The knowledge score is calculated by adding all four items, and a score range from 0 to 4 is generated. A higher score indicates higher knowledge about CMA. HCPs were then classified as having poor knowledge if the score was 0 to 2 points, and 3 to 4 points were classified as a good knowledge level. Likewise, the practice was assessed using a four-item questionnaire. Following the knowledge criteria, HCPs were considered poor practice if the score was 0 to 2 points, and a score of 3 to 4 was regarded as good practice levels.

Descriptive statistics were calculated using SPSS Statistics version 26 (IBM Corp. Released 2019. IBM SPSS Statistics for Windows, Version 26.0. Armonk, NY: IBM Corp). Frequencies and proportions (%) were used to present all qualitative variables, whereas mean and standard deviation were used to elaborate all quantitative variables. The differences in knowledge and practice scores in relation to the HCPs' socio-demographic characteristics were calculated using the Mann-Whitney Z test and the Kruskal-Wallis H test. Statistical collinearity was carried out using the Shapiro-Wilk test and Kolmogorov-Smirnov test. Data follow the non-normal distribution. Therefore, the non-parametric tests were applied. The correlation between the knowledge and practice scores was performed using Spearman's correlation. The statistical significance level was set to less than p=0.05.

Ethical approval was sought from the Regional Ethical Committee of Qassim Region, KSA (607-44-12770). Participants were ensured confidentiality and the freedom to withdraw from the study at any time. Informed consent was taken prior to filling out the questionnaire.

## Results

This study enrolled 124 HCPs. As described in Table 1, 29% were aged between 25 and 30 years old, with more than half (50.8%) being males and 58.1% being of Saudi nationality. Regarding HCPs' specialty, approximately 46.8% were resident trainees, and 37.1% had more than 10 years of experience. In addition, half of them (50%) encountered a child with CMA.

**Table 1:** Socio-demographic characteristics of the HCPs * Medical students and interns were excluded from the analysis. CMA: Cow's milk allergy

Study Data	N (%)
Age group	
<25 years	10 (08.1%)
25-30 years	36 (29.0%)
31-35 years	22 (17.7%)
36-40 years	24 (19.4%)
>40 years	32 (25.8%)
Gender	
Male	63 (50.8%)
Female	61 (49.2%)
Nationality	
Saudi	72 (58.1%)
Non-Saudi	52 (41.9%)
Qualification	
Medical student	07 (05.6%)
Resident	58 (46.8%)
Consultant	10 (08.1%)
Specialist	40 (42.3%)
Interns	09 (07.3%)
Specialty ^(n=108)*^	
Family medicine	39 (36.1%)
General practitioner	30 (27.8%)
Pediatrician	06 (05.6%)
Internal medicine	07 (06.5%)
Dentistry	04 (03.7%)
Not specified	22 (20.4%)
Years of experience	
<5 years	53 (42.7%)
5-10 years	25 (20.2%)
>10 years	46 (37.1%)
Have you ever encountered a child with a CMA?	
No	62 (50.0%)
Yes	62 (50.0%)

In the assessment of HCPs' knowledge of CMA (Table 2), 71.8% of HCPs were correct that CMA could be seen more frequently among toddlers aged between 0 and 2. However, only 8.1% were aware that there was more than a 75% chance that children with CMA could outgrow their allergy, and only 37.1% believed that children could outgrow their allergy between the age of three and five years old. Approximately two-thirds of them (66.1%) knew that intestinal colic, atopic eczema, urticaria, and anaphylaxis are all related to CMA. Based on the above knowledge statements, the overall mean score was 1.83 (0.96), with more than three quarters (77.4%) considered poor levels and only 22.6% considered good knowledge levels.

**Table 2:** HCPs' knowledge of CMA * Indicates correct answer. CMA: cow's milk allergy, IgE: immunoglobulin E, SD: standard deviation

Knowledge Statement	N (%)
In which age group is CMA most likely to be seen?	
0-2 years*	89 (71.8%)
3-5 years	26 (21.0%)
6-11 years	09 (07.3%)
>11 years	0
At what percentage of the children with CMA could outgrow their allergy?	
<25%	46 (37.1%)
25-50%	29 (23.4%)
50-75%	10 (08.1%)
>75%*	10 (08.1%)
No idea	29 (23.4%)
When do children with IgE-mediated CMA usually outgrow their allergy and be able to re-introduce milk into their food?	
After the age of 1 year	34 (27.4%)
Between 3 and 5 years of age*	46 (37.1%)
They will never be able to consume cow milk again for life	15 (12.1%)
No idea	29 (23.4%)
Which of the allergic diseases below may be related to cow's milk protein allergy?	
Intestinal colic	16 (12.9%)
Atopic eczema	10 (08.1%)
Urticaria	02 (01.6%)
Anaphylaxis	04 (03.2%)
All of the above*	82 (66.1%)
No idea	10 (08.1%)
Total knowledge score (mean ± SD)	1.83 ± 0.96
Level of knowledge	
Poor (score 0-2)	96 (77.4%)
Good (score 3-4)	28 (22.6%)

Regarding HCPs' practices toward CMA (Table 3), few were aware of the appropriate diagnosis action for a patient with a suspected CMA (16.9%), while 35.5% were aware of the action in terms of proper CMA management. HCPs' practices toward food formulation among cases with CMA were also poor (hydrolyzed formula: 16.1% and amino acid-based formula: 14.5%), while their practice when referring a patient to an allergy specialist was also deemed poor. Based on the above statements, the overall mean practice score was 1.51 (SD 1.01), with poor and good constituting 83.1% and 16.9%, respectively.

**Table 3:** HCPs' clinical practice toward CMA * Indicates correct answer. IgE: Immunoglobulin E, CMPA: cow’s milk protein allergy, SD: standard deviation

Practice Statement	N (%)
What is the ideal (gold standard) action you would take for a patient with a suspected cow's milk protein allergy regarding the diagnosis?	
Double-blinded oral food challenge*	21 (16.9%)
Skin prick test	25 (20.2%)
Food-specific IgE levels	61 (49.2%)
No idea	17 (13.7%)
What action would you take for a patient with a suspected cow's milk protein allergy regarding the management?	
Treat the allergic reaction that has developed and recommend continuing to use milk	12 (09.7%)
Only remove cow's milk from the diet.	39 (31.5%)
Remove cow's milk from the diet but state that milk products such as cheese and yogurt could be consumed	29 (23.4%)
Completely remove cow's milk and all milk products from the diet of both the infant and the mother*	44 (35.5%)
Which formula food would you give in cases of cow's milk protein allergy?	
Regular formula	28 (22.6%)
Soya-based formula	36 (29.0%)
Hydrolyzed formula*	20 (16.1%)
Amino acid-based formula*	18 (14.5%)
Goat milk formula	22 (17.7%)
When you refer the patient to an allergist specialist?	
If the patient is having multiple other atopia*	20 (16.1%)
If the patient avoided all CMPA-containing food and is still symptomatic*	32 (25.8%)
If doubt about diagnosis*	32 (25.8%)
Other	40 (32.3%)
Total practice score (mean ± SD)	1.51 ± 1.01
Level of practice	
Poor (score 0-2)	103 (83.1%)
Good (score 3-4)	21 (16.9%)

**Figure 1:**
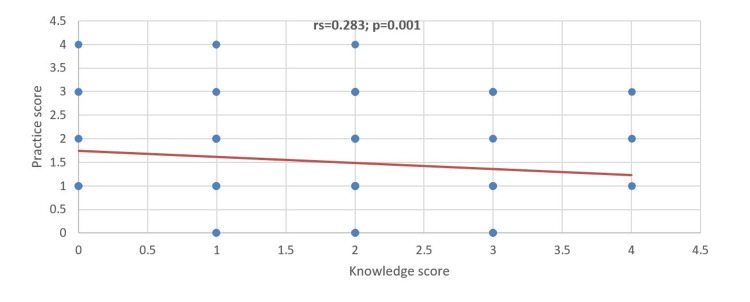
Correlation between the knowledge score and practice score

In Figure 1, it was observed that there was a significant positive correlation between the knowledge score and practice score (rs=0.283; p=0.001).

When measuring the differences in the scores of knowledge and practice in relation to the socio-demographic characteristics of the HCPs (Table 4), it was found that a higher knowledge score was more associated with HCPs who were older (H=7.437; p=0.027), being non-Saudis (Z=2.676; p=0.007), being a consultant/specialist (H=11.583; p=0.003), having more than 10 years of experience (H=12.386; p=0.002), and having encountered a child with a CMA (Z=4.080; p<0.001). On the other hand, a higher practice score was more associated with being a non-Saudi (Z=3.303; p=0.001), being a consultant/specialist (H=9.967; p=0.007), and having more than 10 years of experience (H=10.351; p=0.006).

**Table 4:** Differences in the score of the knowledge and practice in relation to the socio-demographic characteristics of the HCPs ^a^ P-value has been calculated using the Kruskal-Wallis H test. ^b ^P-value has been calculated using the Mann-Whitney Z test. ** Significant at p<0.05 level.

Factor	Knowledge Score (4) Mean ± SD	H/Z-test; P-value	Practice Score (4) Mean ± SD	H/Z-test; P-value
Age group^a^				
≤30 years	1.52 ± 0.84	7.437; 0.024 **	1.24 ± 0.87	5.596; 0.061
31-40 years	1.93 ± 0.95		1.61 ± 1.11	
>40 years	2.13 ± 1.04		1.75 ± 0.98	
Gender^b^				
Male	1.73 ± 0.97	1.310; 0.190	1.41 ± 0.99	1.021; 0.307
Female	1.93 ± 0.82		1.61 ± 1.02	
Nationality^b^				
Saudi	1.63 ± 0.89	2.676; 0.007 **	1.26 ± 0.95	3.303; 0.001 **
Non-Saudi	2.12 ± 0.98		1.85 ± 0.99	
Qualification^a^				
Medical student/intern	1.50 ± 0.97	11.583; 0.003 **	1.31 ± 0.95	9.967; 0.007 **
Resident	1.62 ± 0.88		1.26 ± 0.91	
Consultant/specialist	2.18 ± 0.96		1.86 ± 1.05	
Years of experience^a^				
<5 years	1.57 ± 0.79	12.386; 0.002 **	1.19 ± 0.94	10.351; 0.006 **
5-10 years	1.64 ± 1.04		1.60 ± 0.91	
>10 years	2.24 ± 0.97		1.83 ± 1.04	
Encountered a child with a CMA^b^				
No	1.48 ± 0.88	4.080; <0.001 **	1.37 ± 0.98	1.504; 0.133
Yes	2.18 ± 0.91		1.65 ± 1.03	

## Discussion

This study evaluates HCPs' knowledge and practice levels in children with CMA. According to our results, there was a deficiency in HCPs' knowledge of CMA. Poor understanding of the disease was found in 77.4% of HCPs, and only 22.6% had good knowledge levels. This is consistent with the study done by Erkoçoğlu et al. Their reports show that primary-care physicians' knowledge of food allergies and anaphylaxis was unsatisfactory. Only 47.2% of the participants correctly answered the knowledge-based questionnaire. The authors discussed the aim of improving the standard practice, including the provision of educational programs [13]. Similarly, Faria et al. found knowledge gaps among pediatricians and nutritionists for the treatment of infants with CMA. The investigators emphasized the importance of having a sufficient understanding of the disease, and the lack of knowledge about its treatment may negatively impact the growth and development of these infants [15]. Contradicting these reports, a study conducted in the USA found more than 60% of primary-care physicians correctly answered the questions about knowledge, which was higher than our reports [16]. However, among Turkish pediatricians who underwent occupational training in CMA, they found that occupational education significantly increases the knowledge about CMA among pediatric residents and practicing pediatricians. They assumed that including subjects related to the treatment and management of CMA in the academic curriculum would improve physicians' understanding of the disease [17].

Data in our study indicates that older non-Saudi HCPs with higher qualifications and more years of experience were more likely to demonstrate a better understanding of CMA than other HCPs. Furthermore, although our reports showed that increasing years of experience was associated with increasing knowledge, this did not reflect in the study of Ozkars. According to his reports, no significant association was observed between the group with 1-10 years of experience and the group with 11-30 years of experience regarding the knowledge of CMA [18]. This has been concurred by the study of Erkoçoğlu et al. wherein the difference between years of experience and the knowledge score did not reach statistical significance (p>0.05). In contrast, they found a significant difference between the females and males in terms of knowledge scores, wherein female participants scored significantly higher than their male counterparts [13]. In contrast, gender association with knowledge was not following our results, as the differences between the knowledge score and gender were not statistically significant (p=0.190).

Regarding HCPs' knowledge of the basic facts of CMA, we came to know that even though our respondents exhibited good knowledge about the most affected age group and were aware of the most common allergic diseases associated with CMA, most of them were seen to have a lack of understanding about the percentage in children that could outgrow their allergies, and only 37.1% were aware of the age range where children can usually outgrow their food allergies. Interestingly, half of our respondents had already been involved in managing a child with CMA. In an international study conducted in the Middle East and North African Region which investigated the difference between CMA and cow's milk intolerance, physicians in both regions assumed to have a better perception of the basic concepts of the two subjects as they were able to identify their differences through elimination and challenge tests. Overall, 65% of the respondents were aware of the nutritionally adapted goat's milk formula as a substitute for cow's milk products, and 37% would advise its routine use in infants two years or below [19]. Likewise, Spanish gastroenterologists clearly understood the appropriate diagnosis of children with CMA. They considered the oral challenge to be necessary for the diagnosis of CMA, and most of the respondents were aware of the clinical guidelines for the diagnosis of the allergy [20].

While our subjects presented with poor knowledge, this did not differ from their practices. A considerable proportion of our respondents was regarded as having poor CMA practices (83.1%). Only 16.9% were considered as good. These results stemmed from the specific details of practice statements. For example, only 16.9% knew the appropriate action to diagnose CMA, and while 35.5% could identify the correct management for suspected CMA patients, identifying the proper food formula was found to be unsatisfactory, and even referring patients to an allergy specialist seems undesirable. In one of the studies done in Turkey, epinephrine was the first line of choice of primary-care physicians when managing anaphylaxis. However, only 16.6% were able to give the correct answer about its dosage. The authors concluded that most physicians (98.2%) were interested in attending future meetings about allergic disorders, as their confidence in managing patients with food allergies was basically low (36.7%) [13]. This is almost mirrored by the study of Madrazo et al. Accordingly, they discovered that healthcare professionals were generally confident in managing patients with CMA and LI, and most of them (80%) were also interested in further training to enhance their knowledge of the subjects [12].

Most of our subjects represent poor CMA practices; however, increased practice scores were more frequently seen among non-Saudi consultants/specialists with better years of experience. Furthermore, we also noted a significant positive correlation between knowledge and practice scores. This result suggests that improving HCPs' knowledge will also improve their disease management. To our knowledge, this is the first study in Saudi Arabia to present the differences in practices in terms of HCPs' demographic variables. Albeit, more investigations are required to confirm these findings.

One of the study's limitations was the small sample size. In addition, only primary healthcare doctors were included in the study. The inclusion of doctors from hospitals could show different findings. Further longitudinal studies are required to establish the level of knowledge and practice toward CMA.

## Conclusions

HCPs' knowledge and practice toward CMA were deemed inadequate. However, HCPs who were non-Saudis with a higher level of qualification and more years of experience demonstrated a better understanding and techniques when managing CMA cases than other HCPs. Educational workshops among HCPs may increase the awareness of clinical practice guidelines for the diagnosis and management of children with CMA and help improve clinical outcomes.
